# Cobalt Oxide Synthesis via Flame Spray Pyrolysis as Anode Electrocatalyst for Alkaline Membrane Water Electrolyzer

**DOI:** 10.3390/ma15134626

**Published:** 2022-07-01

**Authors:** Alfonso Pozio, Francesco Bozza, Nicola Lisi, Rosa Chierchia, Francesca Migliorini, Roberto Dondè, Silvana De Iuliis

**Affiliations:** 1 ENEA, C.R. Casaccia, Via Anguillarese 301, 00123 Rome, Italy; francesco.bozza@enea.it (F.B.); nicola.lisi@enea.it (N.L.); rosa.chierchia@enea.it (R.C.); 2 CNR-ICMATE, Institute of Condensed Matter Chemistry and Technology for Energy, Via Cozzi 53, 20125 Milano, Italy; francesca.migliorini@cnr.it (F.M.); roberto.donde@cnr.it (R.D.); silvana.deiuliis@cnr.it (S.D.I.)

**Keywords:** flame spray pyrolysis, electrolysis, AME, cobalt oxide, OER

## Abstract

Nanostructured cobalt oxide powders as electro catalysts for the oxygen evolution reaction (OER) in an alkaline membrane electrolysis cell (AME) were prepared by flame spray synthesis (FS); an AME’s anode was then produced by depositing the FS prepared cobalt oxide powders on an AISI-316 sintered metal fiber by the electrophoretic deposition (EPD) method. FS powders and the composite electrode were characterized by SEM, XRD, and XPS analysis. The electrode showed an increase in the OER catalytic activity in a KOH 0.5 M solution with respect to commercial materials commonly applied in alkaline electrolysis, demonstrating that the flame spray synthesis of nanoparticles combined with the electrophoretic deposition technique represent an effective methodology for producing an anodic catalyst for alkaline membrane electrolyzers.

## 1. Introduction

Hydrogen generation from renewable energy sources (solar, wind, etc.) is one of the most promising options for long-term energy storage [1,2,3]. In particular, water electrolysis is a valid and effective way to produce this energy vector [4]. There are various technologies for water electrolysis that differ in materials, operating temperature, electrolytes, and catalysts. Among the low temperature operating technologies, alkaline electrolyzers (AE) are already industrially widespread and mature [5,6,7]. In particular, membrane-based alkaline electrolyzers (AEM) represent the most interesting technology, thanks to the characteristics of compactness, ease of use, low maintenance, and high pressure performance [8,9]. It has the prerogative to be able to produce hydrogen directly under pressure, which is a fundamental aspect for applications using hydrogen as an energy vector system. Moreover, while other membrane technologies such as PEM need precious metals of the platinum group as electro catalysts, the AE and AEM systems have the advantage of using transition metals such as Co, Ni, and Fe.

The biggest contribution to the cost of hydrogen in this type of technology is the electricity consumption, followed by the cost of the plant. To decrease the hydrogen production price, therefore, it is necessary to both increase the electrolysis efficiency to reduce the electrical consumption and the limitation of the production costs of the components (catalysts, membrane, electrode, etc.). About the efficiency of electrolysis, it must be considered from a thermodynamic perspective that the major limitation of an electrolysis system is related to the slow oxygen evolution reaction (OER) at the anode. Therefore, a high efficiency anode catalyst would go in the direction indicated above. Since AMEs work in an alkaline environment (0.1–1 M KOH), a stable catalyst under alkaline conditions is required. To this purpose, Ni- and Co-based oxides were shown to be effective as OER electro catalysts and an effective way to improve their catalytic efficiency consists of the optimization of their size and surface area. In this paper, we propose a method for the preparation of AEM anodes functionalized by nano structured catalysts, combining the flame spray (FS) synthesis for the production of nanopowders with the electrophoretic deposition technique (EPD) for the deposition of the catalysts on a suitable anode support.

FS represents a well-established, easy to handle, and cost effective technique for the synthesis of nanopowders. Compared to other synthesis techniques such as solid state reaction, spray pyrolysis, or wet methods, it has the advantage of avoiding detrimental steps such as grinding, intensive milling, washing, or heat treatment. Several studies confirm the feasibility of the FSS technique in energetic application, such as the preparation of electrodes for SOFCs [10], electrolyte materials for SOFCs [11], electrodes for batteries [12], or catalysts for EAM anodes [13].

In the perspective of industrial applications, however, a cost effective and easy to scale up technique for the deposition of the electrocatalysts on a suitable anode substrate is required. In a previous work, the authors reported for the first time the preparation of an AEM anode by employing the electrophoretic deposition technique (EPD) for the deposition of commercial cobalt oxide nanopowders on metal fiber stainless steel sintered substrate [14]. This technique (EPD) has the advantage of being easily scalable, low cost, and widely used in industrial applications [15,16]. In the present work, we report the synthesis and the EPD of cobalt oxide nanopowders prepared by the FS method. The powders as well as the AME’s anode obtained are characterized by SEM, XRD, and XPS analysis. Results concerning catalytic activity are presented and compared with measurements obtained with commercial powders.

## 2. Experimental Procedure

### 2.1. Materials and Electrode Preparation

#### 2.1.1. Flame Spray Synthesis

**Figure 1:**
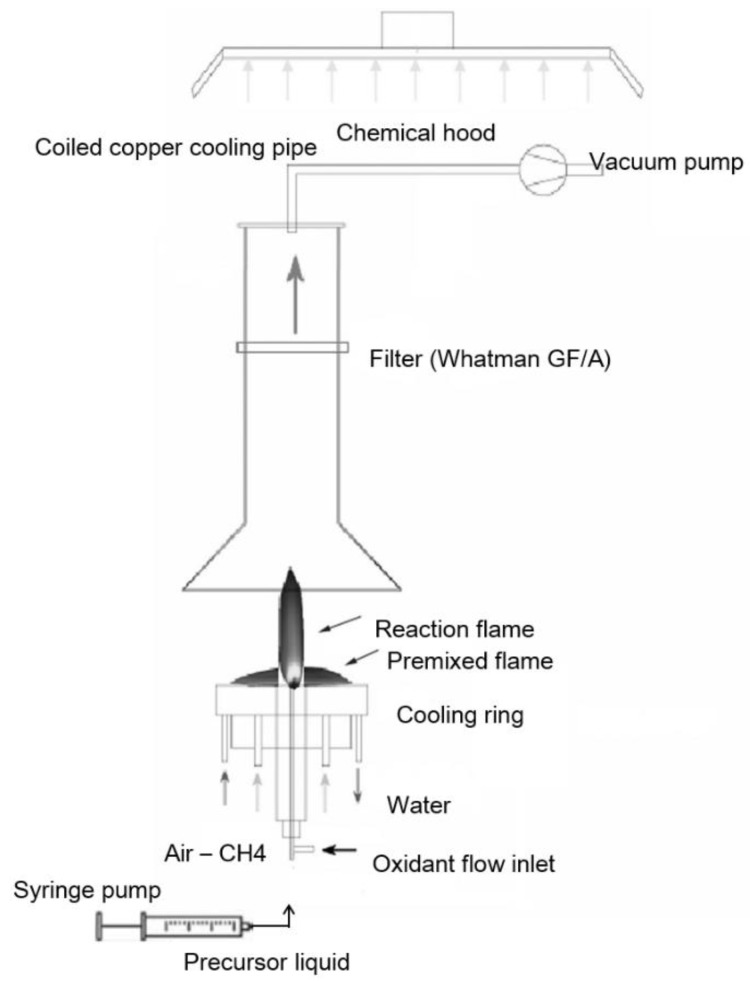
Flame spray pyrolysis apparatus.

For the synthesis of a catalyst, commercial flame spray pyrolysis equipment was used (Np10, ParteQ GmbH). It is essentially an oxygen-assisted spray apparatus, where the precursor is injected coaxially with the pilot flame. The pilot flame is fed with a methane/oxygen mixture to produce a lean premixed flame working close to the stoichiometric condition. The precursor solution flows through the capillary in a stream of O_2_ used as nebulizing gas. The resulting droplets (fuel) start to react with oxygen in diffusion flame conditions (Figure 1). By changing fuel and oxygen flow rate, the experimental conditions of the flame will change (i.e., temperature field, gas velocity, and consequently residence time as well as fuel/oxygen ratio). 

Cobalt(II) nitrate hexahydrate (Co(NO_3_)_2_ × 6H_2_O) dissolved in ethanol (0.3 M) was used as a liquid precursor. The solution was injected at a 4 mL/min feed rate through the spray nozzle by means of a syringe pump and nebulized using a 5 NL/min flow rate of O_2_ stream. Cobalt oxide nanoparticles were produced and collected downstream from the reactor by a vacuum pump system on a paper filter (150 mm diameter).

#### 2.1.2. Electrode Preparation

The AEM anode was produced by depositing the FS prepared cobalt oxide nanopowders on an AISI 316-L (Bekaert) steel substrate having a thickness of 0.51 mm and a porosity of 82%.

In the EPD process, the powders were dispersed in a solution of acetyl-acetone (>99%, Aldrich), deionized water (10 mL L^−1^), and metal iodine (Aldrich, 3 mg L^−1^). The suspension was sonicated for 120 s and allowed to settle for 4 min to let undesired agglomerates deposit on the bottom. The supernatant was then employed for the deposition.

The deposition bath was carried out in a 30 mL Teflon cell. The electrode to be coated and the counter electrode, both made of steel, were placed at a fixed distance of 2 cm and connected, respectively, to the cathode and anode of a PS251-2 (Aldrich) voltage generator. The depositions were carried out at an operating voltage of 40 V for 300 s. After deposition, the electrode was dried and thermally treated in air at 600 °C for 5 h in order to guarantee cohesion between the deposited Co_3_O_4_ particles and to eliminate solvent residues. In this case, the EPD composite electrode was also compared with a commercial NiFe_2_O_4_ (dioxide materials) anode used in several works as a reference standard [17,18].

### 2.2. Surface Analysis

SEM micrographs were acquired with a field emission gun scanning electron microscope Leo 1530 model. XRD measurements were conducted by a RIGAKU Smartlab X-ray diffractometer. XPS analysis was performed with an Escalab MKII equipped with a twin anode, non-monochromated X-ray source. The core level spectra of Co, Fe, C, and O were recorded using Mg (1254 eV) for all sample electrodes before and after exposure to the alkaline cell environment.

### 2.3. Electrochemical Measurements

The electrochemical performance of the anode was verified in a three-electrode cell and in a small electrolyzer at room temperature. The cell with three Plexiglas electrodes was made with a flat platinum counter electrode (area 10 cm^2^) and a standard calomel reference (SCE) through a Luggin capillary positioned as close as possible to the surface of the working electrode. The sample with an exposed active surface of 0.785 cm^2^ was immersed in a solution of 0.5 M KOH and placed parallel to the counter electrode at a distance of 1.5 cm.

Potentiodynamic tests were performed with a 1287 potentiostat (Solartron) at a scan rate of 1 mV s^−1^ in the potential range −0.24 ÷ 1.74 V vs. NHE. Electrochemical impedance spectroscopy (EIS) measurements were performed in the 300 kHz–1 Hz frequency range at open circuit potential (OCP) and with an AC signal amplitude of 10 mV_pp_.

To evaluate the performance of the membrane, a lab-scale 2 cm^2^ electrolyzer was employed, with SS as a cathode and a commercial anionic membrane (Fumasep FAA-3PK-130) as an electrolyte and gas separation system. The cell hardware was made of steel on both cathode and anodic sides. The anodic side can be fed with distilled water or a KOH solution, contained in a 500 mL polyethylene tank using a metering pump (KMS), in PTFE with a flow of 100 mL min^−1^. 

## 3. Results and Discussion

### 3.1. Flame Spray Synthesis of Nanopowders and Their EPD on Anode Substrate

**Figure 2:**
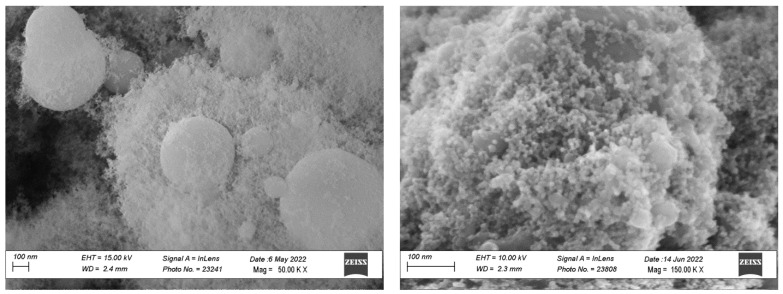
SEM image of synthesized cobalt oxide powder prepared by the flame spray method.

Figure 2 reports the morphology of cobalt oxide nanopowders prepared by FS. The powders show a bimodal size distribution, with spherical particles of submicrometric size surrounded by nanoparticles having a size below 30 nm. The nanosized particles are formed through a gas-to-particle conversion mechanism which consists of the evaporation of the precursors, nucleation in the gas phase, and particle growth. The formation of the large particles can be explained with the existence of the crystal water in the nitrate precursor, which then forms a mixed ethanol/water solvent when dissolved in ethanol: during synthesis, the faster evaporation of ethanol from the droplet leads to the supersaturation and reaction of the precursors on the droplet surface or inside the droplets; the subsequent densification then leads to the formation of the dense large particles [10].

**Figure 3:**
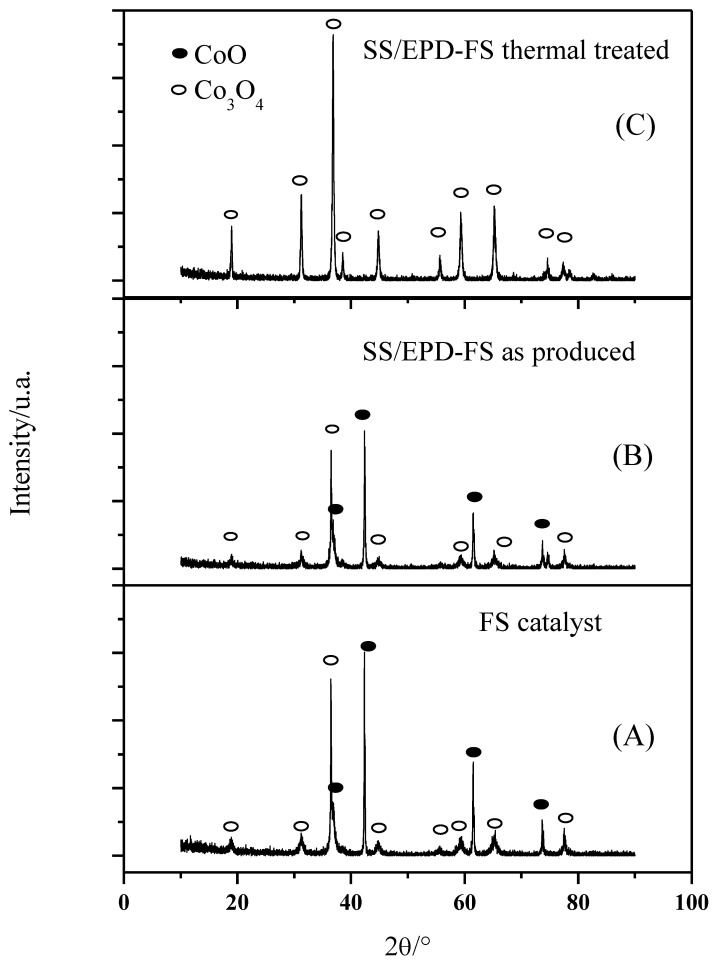
XRD of flame spray catalyst (**A**), after EPD (**B**), and after EPD and thermal treatment at 600 °C in air for 5 h (**C**).

A bimodal behavior of the FS powders is observed also in the XRD phase composition (Figure 3A): the powders exhibit sharp Co_3_O_4_ peaks, which can be associated withthe submicrometric particles, together with broader CoO peaks which are indicative of a nanosized morphology. The average crystallite size of the nanoparticles calculated according to Debye–Scherrer’s equation was 27 nm for the smaller particles, confirming the SEM images. 

As the FS powder has first been EPD deposited on the anode support and then calcined at 600 °C to form an AEM anode, we have investigated the crystallographic behavior of the catalyst with the processing steps. As shown in Figure 3B, the lonely EPD process does not affect the phase composition of the catalyst since the mixed CoO and the Co_3_O_4_ phases have been detected both in the as-prepared powders and in the EPD deposited powders. The following calcination step, however, tends to oxidize the CoO phase to form merely the Co_3_O_4_ phase (Figure 3C). After calcination at 600 °C, however, only the Co_3_O_4_ phase (Figure 3C) can be detected, since the CoO phase tends to form Co_3_O_4_ when heated in air between 600 and 900 °C [19].

**Figure 4:**
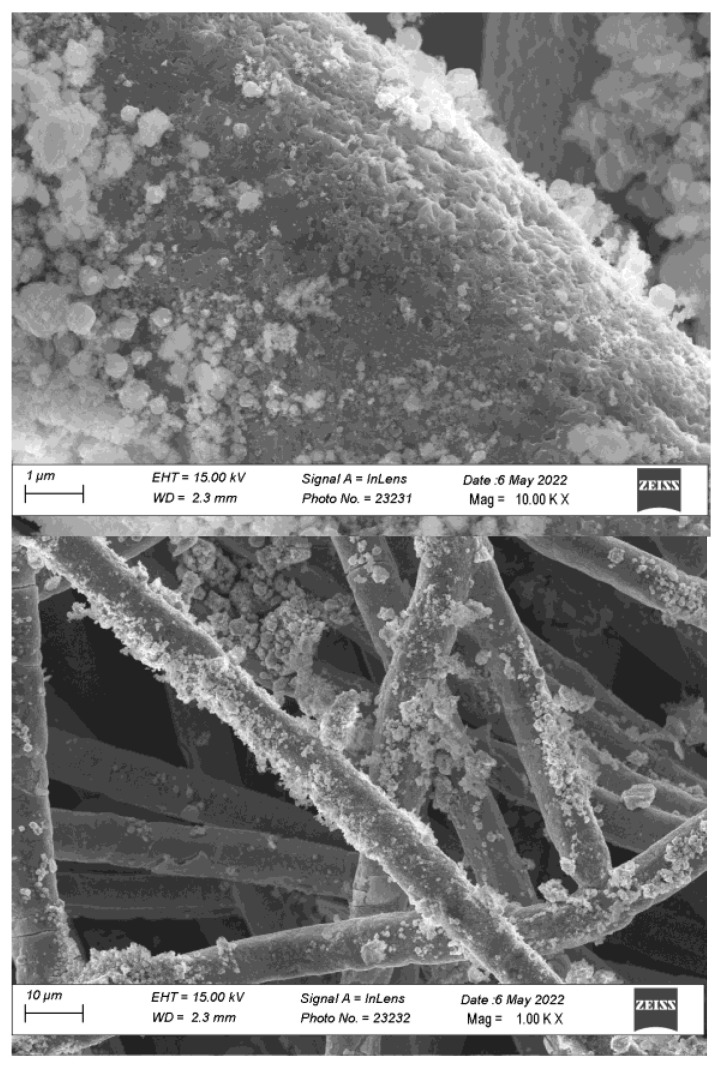
SEM image of an AEM anode obtained by depositing FS prepared cobalt oxide on an AISI-316 sintered metal fiber by EPD followed by sintering at 600 °C in air.

Figure 4 reports the SEM images of the heat treated FS-EPD anode. The anode shows a non-uniform distribution of the catalyst on the fibers, probably as a consequence of the non-homogeneity of the electric field during the EPD process. 

At a more detailed analysis, the FS catalyst still retains its nanostructure after the calcination process, since nanometric particles spread all over the fibers’ surface are visible. The larger submicrometric particles are less evident, because of the sedimentation process before EPD, which selects the heavier particles.

### 3.2. Electrochemical Characterization of Electrodes

The electrochemical performance of the SS/EPD-Co-FS anode was compared with the SS steel support after high temperature heat treatment and with a commercial SS/NiFe_2_O4 electrode. The impedance of the cell was measured before potentiodynamics to verify the ohmic drop and confirm that the observed difference was due only to the anodes showing a constant area surface resistance of 4.3 ohm cm^2^.

**Figure 5:**
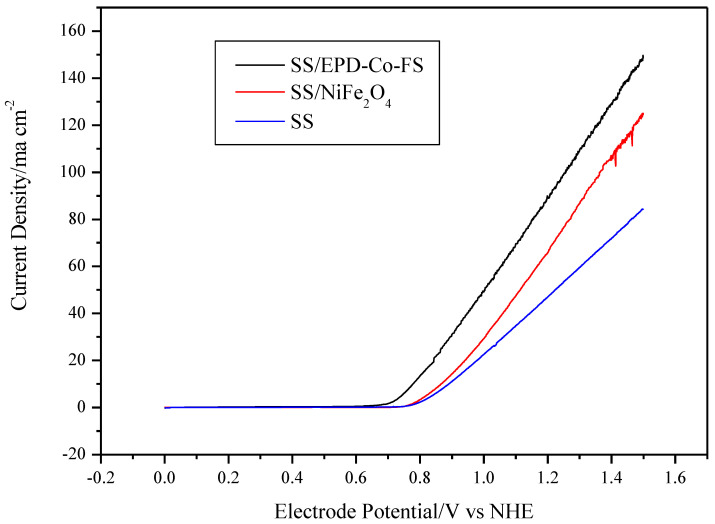
Voltammetries (1 mV s^−1^) in 0.5 M KOH at 25 °C for SS/EPD˗Co_3_O_4_˗FS, SS/NiFe_3_O_4_, and SS.

The voltammetries (Figure 5) show different performances for the three electrodes. The value of the maximum current density follows the trend SS/EPD-Co-FS> SS/NiFe_2_O_4_> SS. The increase in current density at 1.25V vs. NHE for SS/EPD-Co-FS is +85% compared to SS without catalyst and +30% compared to commercial SS/NiFe_2_O_4_. The formation of surface oxides of Fe and Ni with greater catalytic activity for OER [20], caused by the heat treatment on steel, produces an increase in the maximum current of about 21% at 1.75 V compared to NHE [14]; this is probably due to the formation of surface oxides of Fe and Ni with greater catalytic activity for OER [21]. The deposition of Co_3_O_4_ catalyst and the subsequent heat treatment further enhances the current.

The onset potential for O_2_ discharge shows the opposite trend, decreasing from about 0.80 V vs. NHE for SS, 0.78 V for SS/NiFe_2_O_4_, down to about 0.71 V for SS/EPD-Co-FS. Considering the thermodynamic potential for the discharge of oxygen in an alkaline environment (0.401 V vs. NHE, for Equation (1)), the electrode potential in Figure 5, up to this constant, represents the overvoltage for the OER:

(1) $$2OH^{-}\to 1/2O_{2}+2e^{-}+H_{2}O$$

In addition, the Tafel slope and the exchange current density were, respectively, 35 mV/dec and 1.0 × 10^−5^ A cm^−2^ for the SS/EPD-Co-FS and 64 mV/dec and 8.5 × 10^−6^ A cm^−2^ for SS/NiFe_2_O_4_. The better performance of the FS catalyst could be due to the catalyst morphology, which permits easy access of the electrolyte to the entire surface of Co_3_O_4_ and easy escape of the generated oxygen from the electrode. Therefore, the data show that the flame pyrolysis production process is capable of obtaining better results with respect to the commercial one by reducing the overvoltage of almost 80 mV. 

**Figure 6:**
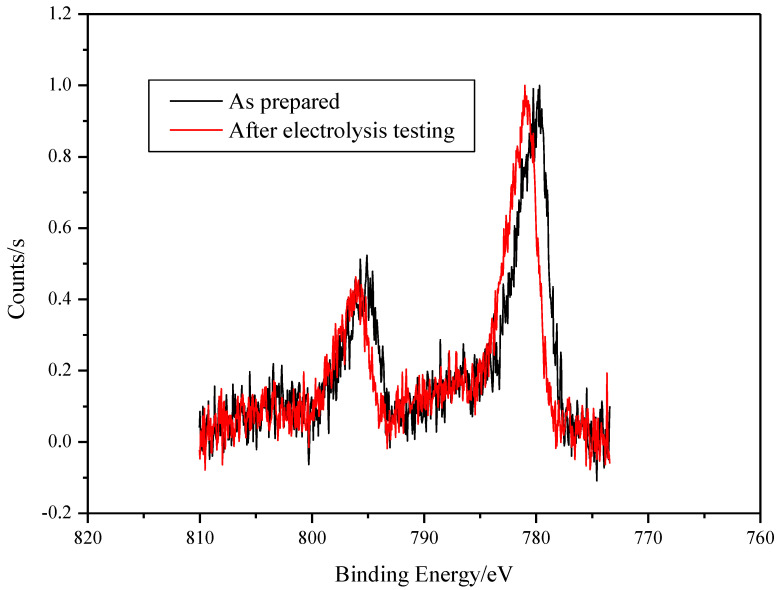
XPS spectra of SS/EPD˗Co˗FS as prepared and after testing electrolysis.

The XPS analysis of the surface on SS/EPD-Co-FS before and after electrolysis measurement shows the presence of Co (Figure 6) oxides [21]. Particularly, the Co 2p peak position and shape confirms that the surface of the as-prepared spray pyrolysis samples is mainly composed of Co_3_O_4_. After the use inside the electrochemical cell, a small shift of the Co 2p peak to higher binding energy (0.8 eV) occurs; following reference [21], the shift indicates that the main surface phase is now cobalt hydroxide Co(OH)_2_.

**Figure 7:**
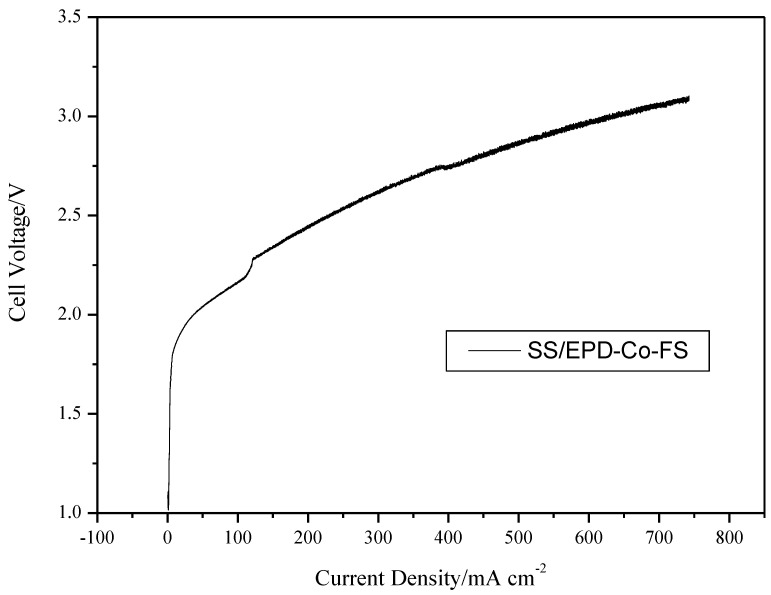
Cell voltage vs. current density for an electrolysis cell with SS/EPD˗Co˗FS, fed with KOH 0.5 M on the anode side at 100 mL min^−1^. Cell temperature 25 °C.

Figure 7 finally shows the polarization results of an electrolysis cell, with the SS/EPD-Co-FP anode, providing direct evidence of the effect of the flame pyrolysis catalyst in obtaining good current density in the range of 2–3 V. In subsequent tests, it will be necessary to check the stability for long time frames and the effect of temperature on the catalytic activity.

## 4. Conclusions

In this preliminary work, a nanostructured cobalt oxide powder was prepared by flame spray synthesis and deposited on sintered metal fiber by EPD. The prepared electrode showed an increase in the OER catalytic activity with respect to commercial materials commonly applied in alkaline electrolysis. The flame spray synthesis of nanoparticles combined with the electrophoretic deposition technique showed to be an effective methodology for producing catalyzed anodes for alkaline membrane electrolyzers.

## Data Availability

The data presented in this study are available on request from the corresponding author.
